# Association of *IL1B -511C/-31T *haplotype and *Helicobacter pylori vacA *genotypes with gastric ulcer and chronic gastritis

**DOI:** 10.1186/1471-230X-10-126

**Published:** 2010-10-27

**Authors:** Dinorah N Martínez-Carrillo, Elvira Garza-González, Reyes Betancourt-Linares, Trinidad Mónico-Manzano, Cuauhtémoc Antúnez-Rivera, Adolfo Román-Román, Eugenia Flores-Alfaro, Berenice Illades-Aguiar, Gloria Fernández-Tilapa

**Affiliations:** 1 Laboratorio de Investigación Clínica, Unidad Académica de Ciencias Químico Biológicas, Universidad Autónoma de Guerrero. Av. Lázaro Cárdenas S/N. Ciudad Universitaria, Colonia Haciendita, Chilpancingo, Guerrero. C.P. 39090, México; 2 Departamento de Microbiología, Facultad de Medicina, Universidad Autónoma de Nuevo León. Av. Madero y Dr. Aguirre S/N, Colonia Mitras Centro, Monterrey N. L. C. P. 64460 México; 3 Unidad Especializada en Gastroenterología Endoscopia. Privada de Jacarandas No. 5, Col. Residencial Bugambilias, Chilpancingo, Guerrero, C.P. 39090 México; 4 Laboratorio de Enfermedades Crónico Degenerativas, Unidad Académica de Ciencias Químico Biológicas de la Universidad Autónoma de Guerrero. Chilpancingo, Guerrero, México; 5 Laboratorio de Biomedicina Molecular, Unidad Académica de Ciencias Químico Biológicas de la Universidad Autónoma de Guerrero. Chilpancingo, Guerrero, México

## Abstract

**Background:**

The association between proinflammatory cytokine gene polymorphisms and gastric diseases related to *Helicobacter pylori *varies by population and geographic area.

Our objective was to determine if the *IL-1B *-*511 T>C *and -*31 C>T *polymorphisms and *H. pylori vacA *genotypes are associated with risk of chronic gastritis and gastric ulcer in a Mexican population.

**Methods:**

We conducted endoscopic studies in 128 patients with symptoms of dyspepsia. We took two biopsies from the body, antrum, or ulcer edge from each patient, and classified our histopathological findings according to the Sydney System. *H. pylori *infection and *vacA *genotyping were accomplished via PCR from total DNA of the gastric biopsies. We confirmed the presence of anti-*H. pylori *serum IgG and IgM in 102 control subjects. In both case subjects and control subjects, the *IL-1B *-*511 T>C *polymorphism was genotyped by PCR-RFLPs and the *IL-1B -31 C>T *polymorphism was genotyped by pyrosequencing.

**Results:**

Sixty-two point seven (62.7%) of the 102 control subjects were *H. pylori-*seropositive. Among the case subjects, 100 were diagnosed with chronic gastritis and 28 with gastric ulcer. We found that 77% of the patients with chronic gastritis and 85.7% of the patients with gastric ulcer were *H. pylori-*positive. The predominant *H. pylori *genotype was *vacA s1m1 *(58.4%) and the most frequent subtype was *vacA s1*. The -*511 TC*, (rs16944 -511 T>C) genotype and the -*511C *allele were associated with chronic gastritis (OR = 3.1, 95% CI = 1.4-6.8 and OR = 3.0, 95% CI = 1.4-6.0, respectively). The subjects carrying -*31T *(rs1143627 -31 C>T) were found to be at a higher risk of having chronic gastritis (OR = 2.8, 95% CI = 1.3-5.8). The *IL-1B *-*511C/-31T *haplotype was associated with chronic gastritis (OR = 2.1, 95% CI = 1.2-3.8) but not with gastric ulcer.

**Conclusions:**

The *H. pylori vacA *genotypes identified herein were similar to those reported for other regions of Mexico. The *vacA s1m1 *genotype was not associated with gastric ulcer. In the southern Mexican population, the *IL-1B -511C *and -*31T *alleles and the -*511C/-31T *and -*511T/-31T *haplotypes are associated with increased risk of chronic gastritis and gastric ulcer.

## Background

*Helicobacter pylori *infection is related to the inflammatory response of the gastric mucosa. While most infected individuals remain asymptomatic, persistent colonization and chronic inflammation increase the risk of developing atrophic gastritis, peptic ulcers, and distal gastric adenocarcinoma [1]. The development of chronic gastritis is the initiating event in the process that leads to stomach cancer. The risk of malignancy increases with severity, chronicity, and duration of the inflammatory process [2,3]. Clinical outcome of *H. pylori *infection is determined by the genetic characteristics of the host and bacteria as well as environmental factors [4]. While *H. pylori *is considered to be a class I carcinogen, it is accepted that some genotypes have greater virulence. The strains that express cytotoxin-associated gene A (CagA) and large quantities of vacuolating cytotoxin (VacA) are most frequently found in patients with peptic ulcers and gastric carcinoma [2,5,6]. It has been observed that *H. pylori vacA s1/m1 *strains produce high levels of the cytotoxin, strains *s1/m2 *produce moderate levels, and strains *s2/m2 *produce little or no toxin [7,9]. The *vacA s1 *subtype is related to higher disease severity and a higher risk of developing ulcers and stomach cancer [5,6,10].

*H. pylori *induce production of IL-1β in the gastric mucosa. IL-1β modulates the expression of other proinflammatory cytokine genes such as TNF-α, IL-2, IL-6 and IL-12, which increase the magnitude of inflammation [11]. The concentration of IL-1β produced by the inflamed epithelium is influenced by two biallelic polymorphisms in positions -511T>C (rs16944) and -31C>T (rs1143627). These polymorphisms are in almost total genetic disequilibrium, and -31 is a TATA-box polymorphism that significantly affects DNA-protein interactions *in vitro*. Thus, these single-nucleotide polymorphisms (SNPs) can modulate production of IL-1β, directly affecting transcription [12,13]. Given that IL-1β is a strong inhibitor of gastric acid secretion and may contribute to dispersion of *H. pylori *from the pylorus to the corpus of the stomach, polymorphisms in the IL-1β gene can be considered a key genetic factor in determining the pattern of gastritis that develops and one risk of malignant transformation [13,14]. The *IL-1B -511T *and -*31C *alleles are associated with high levels of the cytokine and with severe inflammation or stomach cancer, in comparison to -*511C *and -*31T*, which are associated with low levels of IL-1β. This association with *H pylori *infection or stomach cancer has not been significant in all populations [2,4,6,11,13-20].

In combination, bacterium virulence factors and *IL-B *polymorphisms (cagA+/vacA+/IL-1B-511T/IL-RN*2) are associated with severe histological changes in the gastric mucosa of some populations [1,4]. However, the variability of results across populations and geographic areas has been a source of ongoing controversy [2,13,14]. While gastritis, ulcer, and duodenitis constitute the fourth leading cause of disease in the Mexican population [21], the genetic basis for inter-individual variations in inflammatory response and cytokine production, in the context of *H. pylori *infection, has not been well studied in the Mexican population.

Genotyping of *H. pylori vacA *and *IL-1B *polymorphisms could be important in the early identification of individuals at high risk for developing severe gastric diseases. The objective of this study was to evaluate the relationship between *IL-1B *-*511 T>C *and -*31 C>T *polymorphisms and the presence of chronic gastritis and gastric ulcer, and to analyze the relationship of *H. pylori vacA *genotypes with gastric ulcer.

## Methods

### Population

We studied 128 patients subjected to an endoscopic study in the Specialized Unit for Gastroenterology Endoscopy in the city of Chilpancingo, in the state of Guerrero, Mexico, from April 2007 to May 2008. All subjects suffered from functional dyspepsia, epigastric pain, and ulcerative syndrome. Patients who had not received *H. pylori *erradication therapy, and had not been treated with proton pump inhibitors nor with gastric pH neutralizers during the two months prior to the endoscopy were included in the study. We also studied 102 subjects without dyspepsia symptoms, from the same population as the cases, with no history of *H. pylori *infection or gastroduodenal diseases. In both groups, we excluded subjects under non-steroidal anti-inflammatory drug (NSAID) treatment.

Informed consent was obtained from participants or their parents. Participants' eating habits, socio-demographic factors, family history of gastritis or ulcers, alcohol consumption, and smoking habits were recorded via surveys. The study was approved by the Bioethics Committee of the Universidad Autónoma de Guerrero.

### Endoscopy and gastric histology

For each patient, endoscopy was carried out using a video processor and video gastroscope (Fujinon, Wayne, NJ, USA). From each patient, we took two biopsies from the antrum, corpus, or ulcer edge; one specimen was immediately fixed in formalin for histological testing and the other was placed in buffer solution (Tris 10 mM pH 8.0, EDTA 20 mM pH 8.0, SDS 0.5%) for *H. pylori *diagnosis. The biopsies intended for *H. pylori *detection were kept at -20°C until processing. The histological sections were stained with hematoxylin-eosin and evaluated by a pathologist using the updated Sydney System criteria [22]. Endoscopic observation and histopathological confirmation were used to determine patient pathology.

### Serology

Blood samples (5 ml) were taken from all control subjects. The serum was tested for IgG and IgM anti-*H. pylori *by enzyme-linked immunosorbent assay (ELISA; International Immuno-Diagnostics, Foster City, CA, USA) according to the manufacturer's instructions. The sensitivity and specificity of this method are 96% and 97%, respectively. A subject was considered *H. pylori*-positive if we detected at least one of the two antibodies.

### *H. pylori *detection and *vacA *genotyping

Total DNA was extracted from the gastric biopsies of each patient via the phenol:chloroform:isoamilic alcohol technique, after digestion with proteinase K [23].

In the cases, the presence of *H. pylori *was detected by polymerase chain reaction (PCR) for the 16S rRNA gene. We used 150 ng total DNA, 2.5 mM MgCl_2_, 0.2 mM dNTPs (Invitrogen, Carlsbad, CA, USA), 10 pmol of each oligonucleotide, and 1 U Platinum^®^Taq DNA Polymerase (Invitrogen, Carlsbad, CA, USA) in a final volume of 15 μL. The *H. pylori*-positive samples were subjected to detection of subtypes of *vacA *by PCR. For PCR of the signal region (s) and medium (m), we used 300 ng total DNA, 1.5 mM MgCl_2_, 0.2 mM dNTP, 15 pmol of each oligonucleotide, and 1 U Platinum^®^Taq DNA Polymerase in a final volume of 20 μL. The amplification protocols were previously described by Atherton *et al*. and Park *et al*. [24,25]. In each PCR, we used DNA from strain ATCC43504, *vacA s1/m1 *as positive control for *H. pylori *and for *vacA *genotypes. The template DNA was substituted with sterile deionized water in the negative control. All PCRs were conducted in a Mastercycler^® ^Ep gradient thermal cycler (Eppendorf, Hamburg, Germany).

### *IL-1B *-511 T>C and -31 C>T genotyping

We obtained peripheral blood DNA from the control subjects using Miller's technique [26]. Genotyping of the *IL-1B *-*511 T>C *SNP was done via PCR-restriction fragment length polymorphism (RFLP) as previously described [1]. Eight-microliter of PCR product were digested with 2 U *AvaI *(New England Biolabs, Ipswich, MA, USA) at 37°C for 12 h and analyzed in 2% agarose gels. The *IL-1B *-*31 C>T *SNP was genotyped by pyrosequencing, following the methodology described by Pérez *et al*. [27]. Of the 230 samples processed, 12% were subjected to PCR-RFLP for genotype verification.

### Statistical Analysis

We applied X^2 ^or Fisher's exact test to compare frequencies between groups, and analysis of variance (ANOVA) or Kruskal-Wallis tests to compare means and medians, respectively. In the control group, we evaluated the Hardy-Weinberg Equilibrium (HWE) for *IL-1B *polymorphisms. To determine the association of *IL-1B *polymorphisms with chronic gastritis and gastric ulcer, we evaluated binomial and polynomial logistic regression models, and used multiplicative interaction models to study the effect of possible interactions between *H. pylori *and *IL-1B *genotypes and gastric ulcer. We calculated Lewontin's D' statistic for linkage disequilibrium between loci. Two-tailed statistical tests were conducted with a significance level of 5% using STATA 10 software.

## Results

### Histological diagnosis

Of the 128 patients examined, 78.1% had chronic gastritis and 21.9% had gastric ulcer. The majority of cases and controls were women. The average age was 29 years for controls (range 17-61), 46 years for chronic gastritis cases (range 11-80), and 56 years for gastric ulcer patients (range 25-83). There were significant differences among the three groups in age, years of schooling, geographic origin, family history of gastritis or ulcer, and smoking habits (p < 0.05), table 1.

**Table 1:** Socio-demographic characteristics in cases and controls.

		Cases		
			
Characteristic	Controls (n = 102)	Chronic gastritis (n = 100)	Gastric ulcer (n = 28)	p-value
**Age (mean ± sd; years)**	29.8 ± 11	46.1 ± 14.6	56.3 ± 17.1	<0.001^€^
**Gender n(%)**				
**Female**	65 (63.7)	64 (64)	19 (67.9)	0.917 ^**θ**^
**Male**	37 (36.3)	36 (36)	9 (32.1)	
**Education [mean (range); years]**	12 (12-17)	12 (6-17)	6 (0-12)	<0.001^**†**^
**Geographical area n(%)**				
**Chilpancingo**	44 (43.1)	30 (30)	5 (17.9)	0.021 ^**θ**^
**Other municipalities**	58 (56.9)	70 (70)	23 (82.1)	
**Family history of gastritis and/or ulcer n(%)**				0.001 ^**θ**^
**No**	49 (48.0)	39 (39.0)	22 (78.6)	
**Yes**	53 (52.0)	61 (61.0)	6 (21.4)	
**Smoking habit n(%)**				
**No**	39 (38.2)	65 (65)	10 (35.7)	<0.001^**θ**^
**Current smoker or former smoker**	63 (61.8)	35 (35)	18 (64.3)	
**Alcohol consumption n(%)**				
**No**	14 (13.7)	22 (22)	7 (25)	0.211 ^**θ**^
**Consumes or consumed**	88 (86.3)	78 (78)	21 (75)	

^€ ^ANOVA test; ^**† **^Kruskal-Wallis test; ^**θ **^X^2 ^Test; ^Ω ^Fisher's exact test

### *H. pylori *infection and *vacA *genotypes

In 64/102 (62.7%) control subjects anti-*H. pylori *antibodies were detected; 47 control subjects (46.1%) were positive for either IgG or IgM and 17 (16.7%) were IgG+/IgM+. Of the 128 cases, 101 (78.9%) were *H. pylori*-positive, and the prevalence varied by diagnosis. One *vacA *genotype was detected in 85/101 (84.2%) infected subjects and two genotypes were detected in 15/101(14.8%) subjects. The *s1m1 *genotype was detected in 59/101(58.4%) infected subjects, *s1m2 *was detected in 37/101 (36.6%), *s2m2 *in 19/101 (18.8%), and *s1m1/s1m2 *in 15/101 (14.8%) infected subjects. One sample did not amplify for *m *subtypes. Genotypes *s1m1 *and *s1m2 *were predominant in the two groups of cases, table 2. Most, 97/116 (83.6%), typed subtypes were *vacA s1*. The *s1*variant was detected in 22/25 (88%) of the patients with ulcer and 75/91(82.4%) of those with gastritis. Co-infection with *s1m1/s1m2 *genotypes was more frequent in patients with chronic gastritis (18.2%) than in those with gastric ulcer (4.2%).There was not significant association between the *H. pylori s1 *subtype and gastric ulcer (OR = 1.9, 95%CI = 0.29-12.8, p = 0.48) as compared to chronic gastritis. *H. pylori *infection was associated with chronic gastritis (OR = 2.3, 95%CI = 1.2-4.1, p = 0.009) and with gastric ulcer (OR = 4.0, 95%CI = 1.3-12.5, p = 0.016) as compared to controls.

**Table 2:** Genotype and alleles of *H. pylori vacA *frequencies in cases.

Genotypes or alleles	Cases		p-value
		
	Chronic gastritis n(%)	Gastric ulcer n(%)	
Genotypes			
*s1m1*	31 (40.2)	13 (54.1)	0.254^Ω^
*s1m2*	16 (20.8)	6 (25.0)	
*s2m2*	16 (20.8)	3 (12.5)	
*s1m1 y s1m2*	14 (18.2)	1 (4.2)	
Not typed *	-	1 (4.2)	
Total	77 (100)	24 (100)	
Alleles *s*			
s1	75 (82.4)	22 (88.0)	0.551^Ω^
s2	16 (17.6)	3 (12.0)	
Total	91 (100)	25 (100)	
Alleles *m*			
m1	45 (49.5)	14 (58.3)	0.187 ^**θ**^
m2	46 (50.5)	10 (41.7)	
Total	91 (100)	24^♪ ^(100)	

* Signal region *s1*genotype ^Ω ^Fisher's exact test ^**θ **^X^2 ^Test

^♪ ^It was not possible to amplify the m allele in one biopsy.

### *IL-1B -511 T>C *and -*31 C>T *polymorphisms

In the control group, the -*511 T>C *and -*31 C>T IL-1B *SNPs genotypes were in Hardy-Weinberg equilibrium (HWE) (for SNP -511 X^2 ^= 0.500, p = 0.479; for SNP -31 X^2 ^= 0.014, p = 0.905). The genotypic frequencies of the *IL-1B *-*511 T>C *and -*31 C>T *SNPs were significantly different between the patient and control groups (-*511 T>C*, p = 0.015; -*31 C>T*, p = 0.027). For rs16944 (-511 T>C), the frequency of genotype TC was greater in patients with gastritis (60%) and ulcer (46.4%) than in control subjects (38.2%). Genotype CC was the most frequent in patients with gastric ulcer (17.9%). The genotype frequency distribution for rs1143627 (-31 C>T) CT was 57.1% among patients with gastric ulcer, 52% among patients with chronic gastritis, and 41.2% among controls. Genotype TT was more frequent in individuals with gastritis. The *IL-1B -31T *allele was the most frequent among patients with chronic gastritis and ulcer compared to control subjects, table 3. Concordance of results between pyrosequencing and PCR-RFLP genotyping of -31 C>T SNP was 96.4%. Linkage disequilibrium was almost complete between *IL-1B-31 *and *IL-1B-511 *in the case subjects (D' = 0.97; p < 0.001).

**Table 3:** Genotypic distribution and allelic frequencies of *IL-1B *polymorphisms in cases and controls.

		Cases		
			
511 T>C SNP	Controls n (%)	Chronic gastritis n (%)	Gastric ulcer n (%)	p-value
Genotype				
TT	53 (52.0)	31 (31.0)	10 (35.7)	0.015 ^θ^
TC	39 (38.2)	60 (60.0)	13 (46.4)	
CC	10 (9.8)	9 (9.0)	5 (17.9)	
Allele				
T	0.711	0.610	0.589	0.060 ^θ^
C	0.289	0.390	0.411	
**-31 C>T SNP**				
Genotype				
CC	52 (51.0)	31 (31.0)	10 (35.7)	0.027 ^Ω^
CT	42 (41.2)	52 (52.0)	16 (57.1)	
TT	8 (7.8)	17 (17.0)	2 (7.1)	
Allele				
C	0.716	0.570	0.643	0.009 ^θ^
T	0.284	0.430	0.357	

^**θ **^X^2 ^Test ^Ω ^Fisher's exact test

The -*511TC/-31CT *genotypes were more frequent among patients with chronic gastritis (48%) and patients with gastric ulcer (42.9%); in contrast, the -*511TT/-31CC *combination was present in 29% and 32.1% of these groups, respectively. *CT/TT *of *IL-1B *-*31 C>T *SNP was associated with the presence of chronic gastritis (OR = 2.8, 95% CI = 1.3-5.8, p = 0.006) but not with the presence of gastric ulcer, table 4. Adjusting for age, place of origin, schooling, smoking habits, family history of gastritis or gastric ulcer, and *H. pylori *infection, *TC/CC *of *IL-1B *-*511 T>C *SNP was significantly associated with ulcer and chronic gastritis together, as compared to controls (OR = 2.9, 95% CI = 1.4-5.8, p = 0.004). Separating by diagnosis, we found association of -*511TC/CC *with chronic gastritis (OR = 3.0, 95%CI = 1.4-6.3, p = 0.003). The -*511C/-31T *and -*511T/-31T *haplotypes were significantly associated with chronic gastritis but not with gastric ulcer, table 5.

**Table 4:** Association of *IL-1B *polymorphisms with chronic gastritis and gastric ulcer.

SNP	Model	Genotype	Gastritis		Ulcer	
			
			OR (95%CI)	p-value	OR (95%CI)	p-value
-511 T>C	Co-dominant	TT	1.0*		1.0*	
		TC	3.1 (1.4-6.8)	0.004	2.3 (0.7-7.6)	0.175
		CC	2.5 (0.7-8.4)	0.148	5.0 (0.9-29.0)	0.075
	Dominant	TT	1.0*		1.0*	
		TC/CC	3.0 (1.4-6.3)	0.003	2.6 (0.8-8.2)	0.094
						
-31C>T	Co-dominant	CC	1.0*		1.0*	
		CT	2.3 (1.1-5.0)	0.035	2.6 (0.8-8.8)	0.112
		TT	5.6 (1.8-17.6)	0.003	1.6 (0.2-12.6)	0.667
	Dominant	CC	1.0*		1.0*	
		CT/TT	2.8 (1.3-5.8)	0.006	2.5 (0.8-7.9)	0.122

* Reference category: healthy individuals. Models adjusted by age, place of origin, education, smoking habit, family history of gastritis or gastric ulcer, and *H. pylori *infection.

**Table 5:** Haplotypes of *IL-1B *SNPs and their association with gastritis and gastric ulcer.

Haplotype	SNP		Controls	Cases					
				
	-511	-31		Chronic gastritis	OR (95%CI)	p-value	Gastric ulcer	OR (95%CI)	p-value
1	T	C	0.706	0.543	1.0*		0.570	1.0*	
2	C	T	0.279	0.363	2.1 (1.2-3.8)	0.02	0.338	1.3 (0.2-7.5)	0.97
3	T	T	0.005	0.067	16 (1.2-221)	0.04	0.019	ID	
4	C	C	0.010	0.027	3 (0.4-24.6)	0.31	0.073	ID	

ID: insufficient data. * Reference category. Models adjusted by age, place of origin, education, smoking habit, family history of gastritis or gastric ulcer, and *H. pylori *infection.

To determine whether carriers of -*511C *and -*31T *alleles who were infected by *H. pylori vacA s1 *showed higher risk of gastritis or ulcer, the OR was calculated for the patients exposed to *H. pylori vacA s1 *within each group, as compared to those infected with *H. pylori vacA s2*. We did not observe significant association between infection with *H. pylori vacA s1 *with chronic gastritis or gastric ulcer in carriers of the *IL-1B-511C *and -*31T *alleles or their haplotypes (data not shown).

## Discussion

In patients with chronic gastritis, we only found genotypes *vacA s1m1*, *s1m2*, and *s2m2*. However the most virulent allele, *vacAs1*, was the most frequent in patients with both diseases. Our results agree with those reported by other authors and confirm that the *s1m1*genotype is in greatest circulation in Mexico [28-30]. The lack of significant association between *vacA s1m1*with gastric ulcer, as compared to chronic gastritis, may be explained by the presence of multiple factors giving rise to the disease and by the small sample size in our study.

Chronic infection with *H. pylori *induces hypochlorhydria, and this is a critical factor in the development of gastric pathology. Thus, the genetic factors in the host that influence acid secretion can also mediate the clinical progress of *H. pylori *infection. IL-1β is a powerful proinflammatory cytokine that is overexpressed in the presence of *H. pylori *and plays an important role in amplifying the inflammatory response to the infection [1,6,31,32]. The biallelic polymorphisms in positions -31 and -511 of *IL-1B *influence cytokine expression; allele T in position -31 forms a TATA-Box that can potentiate and induce expression of IL-1β [32,33]. It has been reported that the *IL-1B -511T/IL-1B-31C *alleles are significantly associated with the development of hypochlorhydria, *H. pylori *infection, gastritis, and stomach cancer, however the variability of results from studies conducted in various populations remain controversial [33-36].

Interestingly, in our study, the -*511 TC/CC *and -*31 CT/TT IL-1B *genotypes were associated with the presence of chronic gastritis and the presence of gastric ulcer when all cases were grouped (-*511 TC/CC *OR _adjusted _= 2.8, 95% CI = 1.6-5.1; -*31 CT/TT *OR _adjusted _= 2.9, 95% CI = 1.6-5.3). Greater risk of chronic gastritis and stomach cancer has also been reported in Japanese population with the -*511CC *genotype and in Chinese population with the CT genotype [37]. In the Thai population, *IL-1B *genotype -*511CC *is considered a risk factor for stomach cancer [38]. A previous study in a Japanese population showed that subjects with the -*31TT *genotype had a significantly higher risk of *H. pylori *infection (OR= 1.74, 95% CI = 1.15-5.63) than subjects with -*31CT *or -*31CC *genotypes. A similar result was obtained for *H. pylori*-seropositive Japanese-Brazilians (OR -*31TT *= 1.45, 95% CI = 1.02-2.07) [13,39]. In a Korean population, the -*31T *polymorphism was found to be associated with stomach cancer [33]; and in Polish and Western Scottish populations, the -*31T *allele was found to be associated with both *H. pylori *infection and hypochlorhydria as well as a higher risk of stomach cancer [32]. In a Spanish population, García-González *et al*. found that *IL-1B *-*511 C/-31 T *contributed to risk of duodenal ulcer [40].

In our study, the effect of the -*31CT/TT *and -*511TC/CC *genotypes on risk of chronic gastritis was greater when we adjusted for factors such as age, place of origin, schooling, smoking habits, family history of gastritis or gastric ulcer, and *H. pylori *infection. Therefore, the habits and lifestyles of the studied individuals modified the risk of developing chronic gastritis. Such findings support the multifactorial model of gastric pathology that includes the host, *H. pylori*, and the environment [13,36,39]. Given the OR values, it is likely that the same is true for gastric ulcer, but due to the small size of the sample it was not possible to obtain significant associations.

To our knowledge, this is the first study to examine the association of -*511 T>C *and -*31 C>T *SNPs in Mexican patients with chronic gastritis and gastric ulcer. Two prior studies found that the -*31CC *genotype was associated with stomach cancer as compared to the -*31TT *genotype (in the northeast region of Mexico, OR = 7.6, 95% CI = 1.73-46.9; in the southeast and central regions of Mexico, OR = 3.19, 95% CI = 1.05-9.68) [6,41]. These differences might be the result of genomic diversity in the populations across different regions of Mexico. The sub-population in the northern region of Mexico has a greater contribution of European ancestry, while the population of Guerrero has a particularly dominant African ancestral contribution, with a minor European contribution and is genetically closer to the Zapotecs [42].

Chronic gastritis and gastric ulcer are part of the natural evolution to stomach cancer [3,4], and it is possible that a similar phenomenon occurs in Mexico as has been observed in Asian populations, in which the distribution of *IL-1B *genotypes differs between the northern region, where there is a high prevalence of stomach cancer, and the southern region, where there is a low prevalence of stomach cancer [33-35,43].

The information available on *IL-1B *alleles associated with diseases linked to *H. pylori *is controversial. While some researchers have found that *IL-1B *-*511TT *and *IL-1B *-*31CC *are proinflammatory [32,44], others have found, with *in vitro *experiments and with stomach cancer patients infected with *H. pylori*, that the *IL-1B -511C *and *IL-1B -31T *alleles potentiate expression of IL-1β in the gastric mucosa [33,45,46]. The results of our work agree with those of Takagi *et al*. who found that the *IL-1B -511CC*/*IL-1B -31TT *genotypes potentiate cytokine production and are significantly associated with the clinical development of *H. pylori *infection [47].

## Conclusions

Patients with chronic gastritis and gastric ulcer were infected predominantly with *H. pylori vacA s1m1 *genotype and the *vacAs1 *allelotype was the most frequent. In chronic gastritis the *s1m1 *y *s1m2 *genotypes tend to be associated in co-infections.

In the population of southern Mexico, -*511C *or -*31T *alleles and -*511C/-31T *or -*511T/-31T *haplotypes of *IL-1B *increase the risk of chronic gastritis and gastric ulcer.

The results of this study support the hypothesis that the combined effect of lifestyle, infection with virulent genotypes of *H. pylori*, and genetic factors of the host, such as *IL-1B -511C/IL-1B -31T *polymorphisms, can play an important role in development of chronic gastritis and gastric ulcer in the Mexican population of the state of Guerrero.

## Competing interests

The authors declare that they have no competing interests.

## Authors' contributions

All authors read and approved the final study. GFT designed and coordinated the study and wrote the draft. DNMC optimized the PCR conditions, carried out molecular analysis and descriptive analysis of the data, and contributed in preparation of the study. EGG directed pyrosequencing and participated in study preparation. EFA conducted statistical data analysis, RBL recruited patients and carried out the endoscopies on gastric biopsies, TMM conducted surveys and carried out PCR, CAR did ELISA and participated in conducting surveys, BIA participated in study design and data analysis, ARR participated in study design and coordination.

## Pre-publication history

The pre-publication history for this paper can be accessed here:

http://www.biomedcentral.com/1471-230X/10/126/prepub
